# Determination of Lateral Modulation Apodization Functions Using a Regularized, Weighted Least Squares Estimation

**DOI:** 10.1155/2010/635294

**Published:** 2010-05-09

**Authors:** Chikayoshi Sumi

**Affiliations:** Department of Information and Communication Sciences, Faculty of Science and Technology, Sophia University, 7-1 Kioi-Cho, Chiyoda-Ku, Tokyo 102-8554, Japan

## Abstract

Recently, work in this group has focused on the lateral cosine modulation method (LCM) which can be used for next-generation ultrasound (US) echo imaging and tissue displacement vector/strain tensor measurements (blood, soft tissues, etc.). For instance, in US echo imaging, a high lateral spatial resolution as well as a high axial spatial resolution can be obtained, and in tissue displacement vector measurements, accurate measurements of lateral tissue displacements as well as of axial tissue displacements can be realized. For an optimal determination of an apodization function for the LCM method, the regularized, weighted minimum-norm least squares (WMNLSs) estimation method is presented in this study. For designed Gaussian-type point spread functions (PSFs) with lateral modulation as an example, the regularized WMNLS estimation in simulations yields better approximations of the designed PSFs having wider lateral bandwidths than a Fraunhofer approximation and a singular-value decomposition (SVD). The usefulness of the regularized WMNLS estimation for the determination of apodization functions is demonstrated.

## 1. Introduction

A beamformer and a transducer are used in applications such as medical ultrasound (US) imaging, blood flow measurement, tissue displacement/strain measurements, and sonar measurements. For these applications, US beamforming parameters such as US frequency, US bandwidth, pulse shape, effective aperture size, and the apodization function are chosen or selected, and appropriate values are set. In addition, US transducer parameters such as the size and materials used for the US array elements are also chosen. In choosing such settings, the US properties of the target are also considered (e.g., attenuation and scattering). Thus, all of the above parameters must be appropriately chosen and set when considering a system that involves the US properties of the target. In general, such parameters are chosen using the knowledge and experience of an engineer.

Recently, a cosine modulation (LCM) method [1–3] was described that was used for US echo imaging [3, 4] and tissue displacement vector measurements (blood, soft tissues, etc.) [3, 5] using the multidimensional autocorrelation method (MAM) [1], the multidimensional Doppler method (MDM) [1], and the multidimensional cross-spectrum phase gradient method (MCSPGM) [6]. Specifically, for instance, in US echo imaging, a high lateral spatial resolution as well as a high axial spatial resolution can be obtained, and in tissue displacement vector measurements, accurate measurements of lateral as well as axial tissue displacement can be realized. Thus, the use of an optimized beamformer can yield a next-generation US imaging system [3–5].

For the LCM method, a lateral Gaussian envelope cosine modulation method (LGECM) [1–3] was first proposed which uses Gaussian functions in the apodization function, so that a PSF which has a lateral Gaussian envelope can be realized. For the determination of the apodization function, a Fraunhofer approximation was used [1, 7]. The effectiveness of the same LGECM method was also reported by Liebgott et al. [8]. After performing the determination, it was reported that the respective uses of parabolic functions (PAM, i.e., parabolic modulation) and Hanning windows (HAM, i.e., Hanning modulation) instead of Gaussian functions (i.e., LGECM) in the apodization function increased the echo bandwidth and echo SNR without generating ringing in the PSF [3, 5, 9]. PAM and HAM also permitted decreases in the effective aperture size (i.e., channels). These modulations were achieved using knowledge of and experience with US propagation. Specifically, the energy of US transmitted from the feet of the apodization function is lost during the propagation, and the energy transmitted from the main lobes of the apodization function contributes to echo signals. These efforts constituted attempts to break away from the use of the Fraunhofer approximation [3, 5, 9].

Currently, efforts are being made to search for the optimal PSF for both US imaging and displacement vector measurements, and in order to construct a required or designed point spread function (PSF), it is proposed to select the aforementioned beamforming parameters on the basis of linear or nonlinear optimizations [3, 9, 10]. Such optimal settings will enable the construction of the best possible beamformer. By optimizing the apodization function, for instance, lateral resolution will be uniform in the axial direction. In addition, a high echo signal-to-noise ratio (SNR) will also be obtained. In [11, 12] for example, with conventional US imaging (i.e., not with lateral modulation imaging) of a cyst, contrast resolution is optimized by a constrained least squares estimation.

For LGECM used in the simulations in [9, 10], the first optimal determination of the apodization function was performed using the singular-value decomposition (SVD) method [13]. However, for LCM, the choice of the number of the largest singular values to be used with the SVD method was very sensitive to the stability of the determination. That is, a change in the number of only one unit generated a significant instability in the determination. Moreover, the obtained stable apodization function yielded two peaks having the same full width at half maximum (FWHM) which was smaller than that obtained with a Fraunhofer approximation, although obtaining such results was meaningful, that is, two symmetric peaks were obtained in the apodization functions.

In the next trial, in this study, the weighted minimum-norm least squares (WMNLSs) estimation method [14] is used. This also achieves regularization [15, 16] using the conjugate gradient method (CGM) [17] and is used to determine the apodization function for the same PSF [3, 10]. As will be shown by simulations, better approximations of designed PSFs with wider lateral bandwidths are obtained than those obtained by using the Fraunhofer approximation and the SVD method. Finally, conclusions and a discussion are provided together with descriptions of future problems.

## 2. Determination of Apodization Functions for Lateral Modulation Using a Regularized, Weighted MNLS Estimation

For the optimization of target parameters, the beam properties of one element must be obtained in advance using analytical, numerical, or experimental methods as a function of the parameters [9, 10]. That is, the properties of the transducer which is to be used are known.

Beamforming is performed during either the transmission or reception of US, or during both. Thus, conventionally, an apodization function can be obtained by dealing with either the transmission or the reception of US. Alternatively, apodization functions may be determined for both, the transmission and reception of US.

In this study, the simultaneous linear equations in [10] are applied to the regularized WMNLS estimation [3, 9] instead of to SVD [10]. The synthesized, transmitted US beam can be considered as a linear weighted superposition of the beams transmitted from the respective elements with suitable delays for focusing. That is, weighting is realized by using the apodization function. Thus, we obtain the simultaneous linear equations involving the unknown apodization vector **x** [10]

(1) Ax=b,

where **A** is a matrix comprising the US beam values transmitted to a region of interest (ROI) from the respective elements of the US array, and **b** is a vector comprising the designed PSF values in the ROI. Here, we assume that the number of equations in (1) is larger than the number of unknown apodization values, that is, the number of elements in the effective aperture (i.e., these are over-determined equations). However, in any case, for the determination of the apodization function, a least squares minimization is performed on (1) with respect to the unknown vector **x**.

However, note that because the independence of the rows of matrix **A** is low, the vector **x** was stably determined previously by obtaining the inverse of **A** using a singular-value decomposition (SVD) [10]. With SVD, small singular values are disregarded [13]. The method of determination, which is not effective in determining the high-frequency components in vector **x**, is effective in the determination of the apodization function in most cases. That is, such a determination is effective when **x** is smooth and has only low-frequency components; that is, when **x** is not used for very near field imaging/measurements. However, as mentioned above, for LGECM, the choice of the number of the largest singular values to be used was very sensitive to the stability of **x** (the 20 largest singular values were used) and the obtained **x** yielded a worse approximation of the designed PSF than that obtained using the Fraunhofer approximation [10].

In this study, a regularized, weighted least squares estimation was used [3, 9] based on the WMNLS estimation [14] and regularization using penalty terms [15, 16] to solve (1). To judge the quality of the approximated, designed PSF, the shape (e.g., FWHM and the length of the feet) of the PSF is used as a measure. Thus, at each position in an ROI, the least squares estimation should be properly executed using a proper weight, and regularization should also be properly executed using appropriate regularization parameter values to stabilize the solution **x **as was described for shear modulus regularization [16], that is, spatially variant regularization. That is, the use of a large weighting factor (i.e., a large diagonal element for a diagonal weight matrix **W**) and a large regularization parameter, respectively, are used to place appropriate values on the equation and penalty term corresponding to the position. Although we may use the envelope of the designed PSF and the reciprocal as appropriate weight matrices, other weightings may also be appropriately used. Thus, the cost function to be minimized with respect to **x** is expressed as

(2) $$II\left(x\right)={||b-Ax||}_{W}^{2}+\alpha_{0}{||x||}_{I}^{2}+\alpha_{1}{||x||}_{D}^{2}+\alpha_{2}{||x||}_{D^{T}D}^{2},$$

where **W** is a spatially variant weight matrix (i.e., ||**c**||_**W**_^2^ = **c** ^**T**^ **W** ^**T**^ **W** **c** with respect to a vector **c** (**T** is a transposition)); *α*_0_, *α*_1_ and *α*_2_ multiplied by the penalty terms (i.e., from the 2nd to 4th terms) are so-called regularization parameters; **I** is an identity matrix; **D** is a gradient operator; **D** ^**T**^ **D** is a Laplacian operator. Thus, the use of large regularization parameters smoothes the solution **x**. The weighting and regularization must also be properly performed at each modulation position.

The minimization of (2) is performed iteratively by using the conjugate gradient method [17], because the regularized WMNLS estimation achieved with such an iterative method is more stable and the number of calculations is also smaller than with SVD (i.e., the direct method) [13–17], particularly when the ROI is large. For the initial estimate with the iterative method, in order to decrease the required number of iterations necessary to converge to the solution, the apodization function obtained by the Fraunhofer approximation can be used. The simultaneous optimization of other beamforming parameters is described in [18] (e.g., a delay pattern in US arrays by determining a complex apodization function, etc.).

In the next section, the same beam property consisting of one element calculated with Field II [19] was used, as was done with the SVD method in [10], to determine the apodization function for LGECM. When the conjugate gradient method is used, as shown, the use of a very large regularization parameter yields only the initial estimate as a result (i.e., a Fraunhofer approximation). 

## 3. Demonstration of an LGECM Determination

Here, as in [10], the apodization function for LGECM was determined. That is, for a modulation depth *x*, the designed lateral (*y*) PSF (i.e., a Gaussian-type PSF with a lateral modulation frequency *f*_*y*_) is

(3) $$\text{PSF}\left(y\right)=\exp\;\left(-\frac{y^{2}}{2\sigma_{y}^{2}}\right)\cos\;\;\left(2\pi f_{y}y\right).$$

In addition to the same lateral standard deviation (SD) *σ*_*y*_ as that used in [10] (i.e., 0.8 mm), a *σ*_*y*_ value of 0.6 mm was also used under conditions which assumed an US speed of 1,500 m/s; an US frequency of 3.5 MHz; a modulation frequency (*f*_*y*_) 1/*λ* mm^−1^; a modulation depth of 33 mm. The envelope of the US pulse (i.e., the axial PSF) was also Gaussian (correspondingly, the same axial SDs *σ*_*x*_ are used, i.e., 0.8 and 0.6 mm). The transducer parameters used were of element size *λ*; a height of 5.0 mm; the space between the elements was 0.1 mm. The axial sampling interval was 0.0833 mm, and the beam pitch was 0.1 mm. The number of transducer elements used was 401 (i.e., the aperture size was 40 mm), and the ROI used was a rectangular region 7 mm (depth from 30 to 37 mm, 85 points) × 20 mm (lateral width, 201 lines) centered on the modulated point. Thus, in (1), the size of matrix **A** was 17,085 (= 85 × 201) × 401; the sizes of the vectors **x** and **b** were, respectively, 401 × 1 and 17,085 × 1. For the regularization, only the penalty term using the gradient operator as the weight matrix was used (i.e., *α*_0_ = *α*_2_ = 0 in (2)).

**Figure 1:**
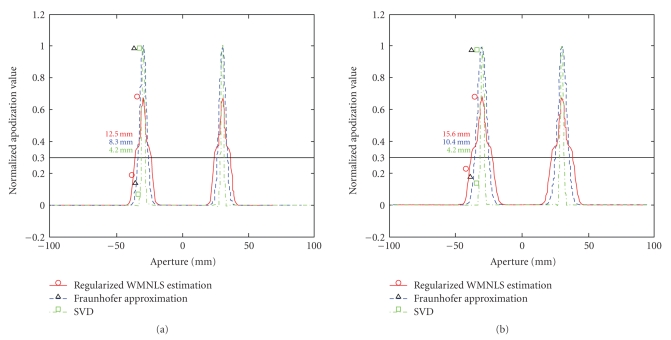
Apodization functions obtained using a regularized WMNLS estimation (solid line with circles), a Fraunhofer approximation (dashed line with triangles), and a SVD (dash-dot line with squares) for designed lateral Gaussian-type PSFs with (a) *σ*_*y*_ = 0.8 and (b) 0.6 mm. The widths of two peaks at the normalized apodization value 0.3 are also shown.

In Figure 1 for *σ*_*y*_ = (a) 0.8 and (b) 0.6 mm, the apodization functions determined with the regularized WMNLS estimation together with those determined by the Fraunhofer approximation and SVD are shown. For *σ*_*y*_ = 0.8 mm (Figure 1(a)), the same apodization functions obtained with the Fraunhofer approximation and SVD are shown in [10]. As shown, the two peaks in the apodization functions obtained with the regularized WMNLS estimation (solid line with circles) have widths larger than those obtained with the Fraunhofer approximation (dashed line with triangles), although when using the SVD (dash-dot line with squares), those obtained have widths smaller than those obtained with the Fraunhofer approximation. For instance, for *σ*_*y*_ = 0.8 mm, the widths at the normalized apodization value 0.3 are, respectively, 12.5, 8.3, and 4.2 mm. The widths are also depicted in Figure 1 together with those for *σ*_*y*_ = 0.6 mm. Thus, the largest lateral bandwidths for the PSFs can be obtained with the regularized WMNLS. 

**Figure 2:**
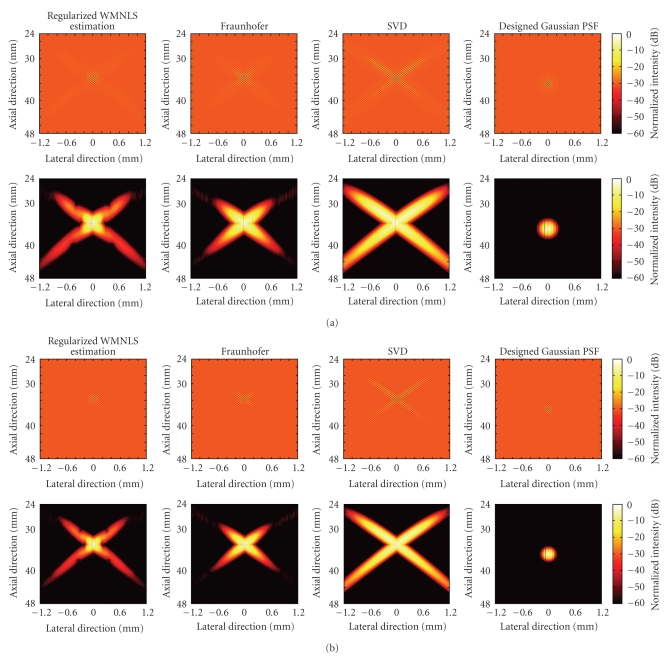
Designed Gaussian-type PSFs and PSFs obtained using apodization functions obtained with a regularized WMNLS estimation, a Fraunhofer approximation, and SVD for (a) *σ*_*y*_ = 0.8 and (b) 0.6 mm.

**Figure 3:**
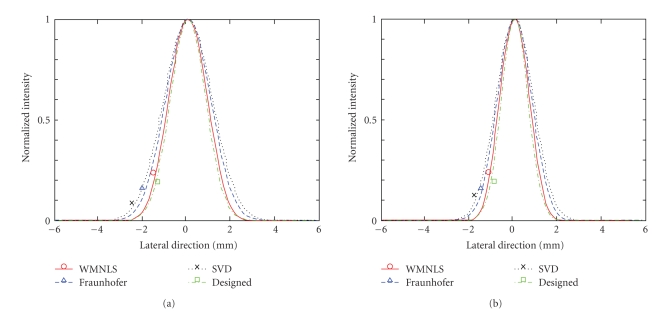
Lateral intensity profiles of a designed PSF (dash-dot line with square) and PSFs obtained using apodization functions obtained with a regularized WMNLS estimation (solid line with circle), a Fraunhofer approximation (dashed line with triangle) and a SVD (dotted line with cross) for (a) *σ*_*y*_ = 0.8 and (b) 0.6 mm.

For the respective values of *σ*_*y*_ = 0.8 and 0.6 mm, the images of the PSFs (one way) and the intensities obtained using the apodization functions determined by the regularized WMNLS estimation, Fraunhofer approximation and SVD are shown in Figures 2(a) and 2(b) together with those of the designed, original PSFs. In addition, the corresponding lateral intensity profiles of the PSFs are shown in Figures 3(a) and 3(b) (the solid line with a circle was obtained with the regularized WMNLS estimation; the dashed line with a triangle was obtained with the Fraunhofer approximation; the dotted line with a cross was obtained with the SVD; the dash-dot line with a square is the designed one). As shown for both values of *σ*_*y*_, PSFs obtained with the regularized WMNLS estimation are better than those obtained with the Fraunhofer approximation. That is, better approximations for the designed PSFs are obtained. In contrast, the approximations obtained with the SVD are worse than those obtained with the Fraunhofer approximation. Also the normalized intensities larger than –20 dB (Figures 2(a) and 2(b)) are compared. Apparently, PSFs obtained with the Fraunhofer approximation and SVD show the two crossed beams. Although not shown, the same results were obtained with regularized WMNLS estimations using the respective designed PSFs and their reciprocals as weights. For the designed PSF, the weightings with a least-squares minimization were not effective.

**Figure 4:**
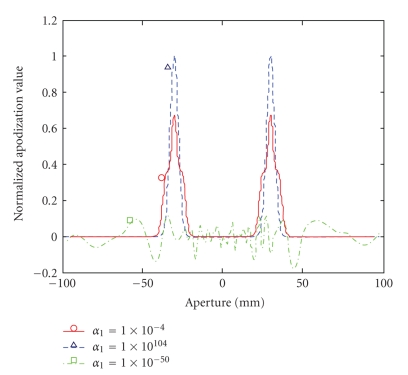
Apodization functions obtained for *σ*_*y*_ = 0.8 mm with a regularized WMNLS estimation using different regularization parameter *α*_1_ values: 1 × 10^−50^ (dash-dot line with square, unstable), 1 × 10^−4^ (solid line with circle, corresponding to the stable apodization function shown in Figure 1(a)), and 1 × 10^104^ (dashed line with triangle, the same as that obtained with a Fraunhofer approximation shown in Figure 1(a)).

Figure 4 shows the apodization functions obtained, when *σ*_*y*_ = 0.8 mm, with the regularized WMNLS estimation using different regularization parameter *α*_1_ values, that is, 1 × 10^−50^ (dash-dot line with a square), 1 × 10^−4^ (the solid line with a circle corresponds to the stably obtained apodization shown in Figure 1(a)) and 1 × 10^104^ (dashed line with a triangle). As shown, the small *α*_1_ still yielded an unstable apodization whereas *α*_1_ larger than 1 × 10^104^ yielded the same apodization as that obtained with a Fraunhofer approximation (also shown in Figure 1(a)), that is, the initial estimate of the conjugate gradient method. *α*_1_ values from 1 × 10^−40^ to 1 × 10^104^ invariantly yielded almost the same apodization functions. The regularization using *α*_0_ was not effective for stabilizing the determination (i.e., not properly smoothed); whereas that using *α*_2_ resulted in almost the same determination as that using *α*_1_.

## 4. Conclusions and Future Problems

For Gaussian-type PSFs (i.e., LGECM), the regularized WMNLS estimation yielded better approximated PSFs having wider lateral bandwidths than the Fraunhofer approximation and the SVD method. The usefulness of the regularized WMNLS method for defining apodization functions was demonstrated. The effectiveness of the spatially variant weightings and regularization will be specifically reported elsewhere. 

Moreover, it has recently been reported that PSFs having envelope shapes of Akaike window, power functions, and new windows which were developed by changing the Hanning window used in the Turkey window by the Akaike window or power functions are desirable in the sense that a wider bandwidth and a higher echo SNR can be obtained than with a Gaussian-type PSF [20]. The feet of the PSFs can also be truncated. Moreover, the effectiveness of a nonlinear optimization on the construction of an apodization function is also shown to result in a better approximation of desirable PSFs (the feet of the two peaks in the linearly optimized apodization function are truncated) [21].

In conventional US imaging (i.e., not modulation imaging) of a cyst, contrast resolution is optimized with another least squares estimation [11, 12]. Here, similarly, targeted US properties will also be used in the construction of PSFs such as frequency-dependent attenuation. Such a method of construction will be reported elsewhere together with the simultaneous determination of multiple parameters [18]. To determine the size and materials for an optimal US element, nonlinear optimization will also be performed. Such constructions will be performed under conditions in which transducers have physically finite aperture widths and various shapes. Thus, for practical applications in a next-generation US imaging system, PSFs that yield the highest-quality US imaging and the most accurate measurements of tissue motion and blood flow (such as displacement vectors and strain tensors) will be developed in the near future. Spatially uniform quality and accuracy will also be realized.

Specifically, for instance, in US imaging, a spatial resolution of less than 3 mm is currently required to overcome the clinical limitations in conventional digital US imaging equipment. Accurate 3D US imaging, 3D tissue motion measurements (3D blood flow vector, tissue strain tensors, etc.), and 3D shear modulus reconstructions [3–5] using a 2D US array [1] and 3D displacement vector/strain measurements will also be achieved in real time as low-dimensional measurements/reconstructions [22] by choosing a narrow 3D ROI. That is, the demonstrated determination of a 1D apodization function can be easily extended to 2D functions. LCM makes it possible to attach an US transducer to the target body in order to achieve the measurements and reconstructions without considering the direction of the target motion. That is, LCM permits freehand measurements and reconstructions in addition to dealing with uncontrollable target motions due to heart motion or pulsation, and with deeply situated tissues which cannot be accessed from the body's surface.

Such LCM methods can also be used in US harmonic imaging and measurements as well as in radar applications [3]. These determinations may also enable new aspects of super-resolution imaging using inverse filtering [3]. Optimal beamforming (LCM, etc.) can also enable the use of effective high intensity focused ultrasound (HIFU) [23] with a high lateral resolution [3]. Such high intensity ultrasound can also be used as a radiation force (ARF) [24, 25] for the imaging of shear waves or treatments. The use of a suitable receiver for HIFU and ARF will also be effective [3]. The evaluation of the newly developed PSFs will also be performed by reconstruction of the mechanical source or thermal source using the proposed differential-type inverse methods (e.g., [26, 27]). Thus, beamforming parameter determinations will also be used to develop a spatially uniform efficiency and accuracy for treatments. Efforts will also be made to determine the high-frequency components in an apodization function for a very near field.

Thus, efforts to develop new US diagnosis/treatment systems using proper beamforming and various methods of computational imaging are currently underway.
